# Incorporating husband’s influence in estimating unmet need for contraception using modified revised algorithm

**DOI:** 10.1371/journal.pone.0357392

**Published:** 2026-08-31

**Authors:** Gerald Mbuthia Mahuro, Murungaru Kimani, Andrew Kyalo Mutuku

**Affiliations:** 1 Kenya School of Government, Nairobi, Kenya; 2 University of Nairobi, Nairobi, Kenya; King’s College Hospital NHS Foundation Trust, UNITED KINGDOM OF GREAT BRITAIN AND NORTHERN IRELAND

## Abstract

Unmet need for contraception is defined as the proportion of exposed fecund reproductive age women desiring to limit fertility but who are contraceptive nonusers, and it has invaluable contributions to population dynamics policies besides engendering fertility discourses. Aligning with the continuous evolution of conceptualization, the fluidity of the robust measurement model was indispensable, stemming from a formula that identified a pool of reproductive age women in irrational situations, discrepant behavior, “KAP-Gap” and unmet need for contraception. Extant fertility literature suggests the possibility of some reproductive age women classified as having unmet need but genuinely have “no unmet” need. This study proffers that exposed women whose husbands have a virility issue attendant to genetic, health, social, and lifestyle factors may obliviously forgo birth control methods on the perception of reduced pregnancy risk. Upon incorporating husbands’ virility loss, this study generated a modified “Revised” algorithm and utilized 2014 Kenya demographic and health survey data to select 8,710 reproductive age women who met fertility criteria. Using Stata ® the modified “Revised” algorithm estimated unmet need for contraception at 16.4 percent, lower than national estimates by 1.1 percentage points, implying 6.6 percent overestimation. The “modified” estimates were highest among adolescents, rural dwellers, and residents of Northeastern region but decreased with increasing education and household wealth. This study has generated a modified “Revised” algorithm, confirmed overestimation of “unmet need” for contraception, incorporated overlooked husband’s virility loss, and proved reclassification of women from “unmet need” to “no unmet need.” These results, therefore, endorse incorporation of virility loss as a reason exuded by exposed reproductive age women for abstaining from birth control methods.

## Introduction

Measurement and understanding of the aspects attendant to unmet need for contraception (UNC) has evolved to be an important area of demographic research due to its invaluable implications to policies regarding population dynamics and fertility discourses [1]. Unmet need for contraception refers to the proportion of exposed fecund women of reproductive age (WRA) within ages 15–49 years, who are desiring to reduce their fertility by either limiting or postponing childbearing despite being contraceptive nonusers [1–3]. Complimentarily, [4] summarized the concept of unmet need for contraception as the difference between the demand and need for contraception, while, [2] described unmet need for contraception as the discrepancy between woman’s fertility desires and contraceptive behavior. Assuredly, a plenitude of literature postulates that estimates of unmet need for contraception is a representation of a pool of women who want to practice contraception but are letdown by diverse intrinsic reasons besides known inadequacies in access to family planning supplies and services [2].

The concept of unmet needs for contraception is not without skepticism that emanated from divergent views, including inconsistent behavior of woman’s pregnancy prevention, underestimation of exposure to the risk of pregnancy, unpredictability of future use of contraceptives, besides inherent measurement and conceptual complexities [2–5]. Interestingly, the unmet need for contraception is an invaluable and widely used concept for measuring how populations’ contraception needs are met, and reveals the size of the potential unfulfilled demand for family planning [2,6]. Several studies have concluded that, reducing unmet need for contraception is responsible for enhancing social, cultural and economic autonomy of women through attainment of preferred family size, improved livelihood, and reduced lifetime fertility [7,8].

The impact of UNC on fertility decline is multipronged because improved contraceptive use is associated with a decline in unmet need for contraception [1] whereas increased contraceptive use is a known major contributor of fertility decline [2,4]. Based on this symbiotic relationship, unmet need for contraception depicts a negative relationship with contraceptive use, and therefore UNC is construed as a proxy measure to reduced lifetime fertility. This has led to extensive and careful examination of unmet need for contraception to the extent of attaining equal stature with contraceptive use. This has elevated UNC to be considered as: A cardinal global justification for increasing resource allocation; evaluating Country’s family planning policies and programs on meeting populations’ felt need; monitoring achievement of sustainable development goal 3.7.1; a hypothetical link between population growth concerns and women’s ability to practice reproductive rights at own volition; and, a valuable link between human rights and feminist approach on matters fertility regulation and demographic dividends [2–4,9].

Understandably, the current ‘Revised’ algorithm is widely accepted as the standard approach in estimating unmet need for contraception, culminating into classifying reproductive age women into four categories of; met need, unmet need, no unmet need and infecund/ menopausal. In this context, [10] argued that there is a possibility of existence of a pool of exposed women of reproductive age who have been classified as having unmet need but they genuinely have no unmet need for contraception. This study corroborates this argument by proposing that there exists a pool of exposed women of reproductive age whose husbands suffer virility issues, thereby portending reduced risk of conception, notwithstanding coital interaction. Appreciating the multi-decade efforts geared towards improving this “Revised” algorithm which was declared by scholars as “irreplaceable in unforeseeable future” [2], scholarly scrutiny is indispensable. In extending this discourse, this study incorporates the overlooked husbands’ effect by generating a modified “Revised” algorithm that provides estimates of unmet need for contraception that are expected to be lower, therefore proffering overestimation of KDHS 2014 estimates.

The husbands’ effect in this context is the diminished fecundity that may be attendant to loss of virility, which medically emanates from genetic, health, social and lifestyle factors. The genetic factors include; azoospermia- No measurable sperm from the fluid ejaculated during orgasm (semen) and this is prevalent in one percent of all men [11], aspermia – complete absence of semen from ejaculate – dry ejaculate [12], undescended testicles, and other genetic defects). The health problems may include diabetes, testicular or prostate cancer, sexually transmitted infections, chronic disease whereas the social problems include stress, depression and hormonal imbalance. Finally, the lifestyle factors include habituation, asexual and trauma [13–15]. Such reasons expressed by a pool of exposed woman of reproductive age as a justification for not using contraception despite having coital interaction are usually analyzed using the “Revised” algorithm as “unmet need” for contraception. In essence, women who are in such situations have a high likelihood of developing a genuine feeling of “superficial protection” and thus portend a reduced risk of pregnancy. To this end, this pool of exposed reproductive age women genuinely qualify to be considered as having “no unmet need” for contraception. This consideration appraises the fact that some exposed fecund women of reproductive age being consciously aware of reduced/ no risk of conception attendant to husbands’ virility issues, may obliviously decide to abstain from any contraception use. This study therefore operationalizes this argument by developing another constraint for inclusion in the “Revised” algorithm to develop the modified “Revised” algorithm that generates “Modified” estimates of unmet need for contraception. Upon incorporating this husbands’ virility aspect in the “Revised” algorithm to cater for overestimation, the emerging evidence will guide in designing the UNC as best estimator of total fertility rate besides allocating optimal resources for family planning programs, globally.

## Evolution of measurement procedure

As the conceptual definition of unmet need for contraception gained attention in fertility discourses thus becoming confounded, concurrently, demographers, family planning practitioners and researchers developed ideas bespoke precise measurement procedures. The inaugural quantification was made by [16] who used data from three Knowledge, Attitudes and Practice (KAP) surveys conducted in Taiwan in 1965 (KAP-I), 1967 (KAP-II) and 1970 (KAP-III). In each of these KAP surveys, a probability sample was randomly selected to represent all married women of reproductive age as survey respondents. In attempting to conceptualize the formulae used in computing this pool of women, let xi be number of married women who are contraceptive nonusers, yi be number of married women who want no more children, zi be married women aged 22–39 years and ∩ be the intersection of elements that belong to unique sets as stipulated in Set Theory. The proportion of married women who were not using contraception and who wanted no more children can be derived by the general formulae $\left(\frac{xi\cap yi}{zi}\right)$. Further, the pool of women “who were married who were contraceptive nonusers, who wanted no more children”, can be simplified as $\left(\frac{xi}{yi}\right)$, and thus termed as being in an irrational situation.

Further examination of women in “irrational situation” was done by [17] who extended the analysis by adding two more KAP surveys that were conducted in Taiwan in 1971 and 1973. The estimation used a unique time series technique based on the concept of a function of two components that included percent of “women who wanted no additional children and percent who were contraceptive nonusers” among those who wanted no additional children. Similarly, to operationalize this formulae, let xi be number of married women who were contraceptive nonusers yi be number of married women who wanted no additional children, zi be married women aged 20–39 years. The percent among those who wanted no additional children who were contraceptive nonusers is given by $\left(\frac{xi}{yi}\right)$. Further, the percent “who wanted no additional children and were not practicing contraception” can be given by $\frac{yi\cap xi}{zi}$. These results depicted a difference between “need” and “use” of contraceptive services, prompting to term such pool of women as exhibiting “discrepant behavior”. Borrowing from psychology literature, which defines the discrepancy between need and use as gap, the resulting estimates were named “KAP-Gap”.

In extending the estimation of KAP-gap, the first attempt to measure unmet need for contraception was done by [18] in 1978 who extracted data without making any arithmetic computations or multivariate analysis from the published First Country Reports (FCR). The FCR was developed from data collected using World Fertility Survey (WFS) conducted in five Asian Countries namely; Korea (1974), Malaysia (1974), Pakistan (1975), Nepal (1976) and Thailand in 1975. To operationalize this computation, let xi be number of married women who were contraceptive nonusers, yi be number of married women who wanted no more children, zi be women aged 20–45 years exposed to risk of conception, zi* be all married women inclusive of currently pregnant and infecund. Five possible simulations were proposed which include; the first measure computed the percent of exposed women “who wanted no more children” and can be summarized as $\left(\frac{yi}{zi}\right)$. The second measure calculated the percent of “exposed women not using any method or effective method” amongst exposed women, which can be summarized as $\left(\frac{xi}{zi}\right)$. The third measure was computed as “of exposed women who wanted no more, the percent not using any method (efficient)” which can be simplified as $\left(\frac{xi}{yi}\right)$. The fourth measure was measured as “of all exposed women, percent who wanted no more children and were not using contraceptive method (efficient)” which can be simplified as $\left(\frac{xi\cap yi}{zi}\right)$. Finally, the fifth measure was computed as of all currently married (currently pregnant and infecund) percent “who were exposed and wanted no more children and were not using contraceptive users”, simplified as $\left(\frac{xi\cap yi}{zi*}\right).$

There arose a methodological issue on measurement of unmet need for contraception due to exclusion of pregnant or amenorrheic women and the need for spacing childbearing which was identified by [19]. The solution involved applying stringent restrictions through a series of inclusion and exclusion of various variables, and the proposed arithmetic formulae was transformed into an algorithm. Considering exposed women of reproductive age as the denominator, the algorithm incorporated 12 alternative measures with the numerator having variables regarding women’s status of: Fecundity; breastfeeding; type of contraceptive used and pronatalism, culminating into 12 alternative measures. In exploring the algorithm, let x1 be wanted no more children, x2 be desired number of children is less than actual, x3 be fecund but not pregnant, x4 be not breastfeeding, x5 be not using effective method, x6 be not using any method, zi be all currently married women. Using the WFS data, the simplest estimate of unmet need using measure 1 averaged to 40.3 percent and can be simplified as $\left(\frac{x\text{1}\cap x\text{4}}{zi}\right)$, and computed as the proportion of married women who wanted no more children and were not using an effective contraceptive method against all currently married women. Other attenuations considered; exclusion of women who were not pregnant or infecund, which reduced the estimate to 14.1 percent (measure 4), exclusion of women who were within one year of breastfeeding, thus reducing the estimate to 9.0 percent (measure 8) and further exclusion of stringent family size desires which reduced the estimate to 7.2 percent (measure 9). Despite recommending measure 4 and 9 as most subtle and coherent estimates, the authors concluded that there was no “best measure” for unmet need for contraception. Further, they recommended that existence of confounding factors such as program nature, implementation fidelity and funding levels must be considered when deciding which measure to adopt.

Further, [20] noted that focus on potential demand for contraception has stimulated discourses on the high proportion of women especially in developing countries who indicated they wanted no additional children and were relatively low contraceptive users. This was exacerbated by the fact that some women had annual live birth, which was a satisfactory evidence of fecundity and failure to space or limit childbearing, a precursor to unintended pregnancy that forms bulk of unmet need. This dissertation inferred for inclusion in the original algorithm, women who were temporarily not using contraceptives by being pregnant, breastfeeding or in postpartum amenorrheic status as they may/will require contraception once, their protection status elapses. In addition, to weed out unintended pregnancy in-order to cater for desires for spacing or limiting children, the “wantedness” of current pregnancy and timing of their next pregnancy were examined. These suggestions formed a dynamic model that incorporated time as a factor and ability for women to rejoin the exposed group once their natural protection elapsed since they were frequently excluded analytically when estimating unmet need for contraception. This dynamic model widened the applicability of the measurement of unmet need for contraception through inclusion of birth spacers and method failure as prime cause of “unwanted” pregnancy leading to women giving birth each year. Further, these findings implied that, there existed a substantial unexploited potential demand for contraception that could not be accounted for by birth spacers.

Intelligibly, [21] devised a new “current” measure for estimating unmet need for contraception by incorporating pregnant and recently pregnant or amenorrheic women whose pregnancy was unintentional (mistimed or unwanted) and delineated into spacing and limiting child births. This was accomplished by combining numerous identified variables from earlier measures by [19] “current status” model and the “dynamic” model by [20] to develop an “Original” algorithm for estimating unmet need for contraception. The “Original” algorithm which was implemented since approximately 2003, used 15 different questions from demographic and health survey (DHS) questionnaire to classify women into ten mutually exclusive subsets of; using contraception to space, using contraception to limit, unmet need to space, unmet need to limit, spacing failure, limiting failure, desires a birth within two years, never had sex, no sex/ want to wait, or infecund.

The continued refinement to improve the understanding of unmet need for contraception complicated the measurement model culminating into all fertility related surveys lacking comparability across countries and over time, and consistency during calculation and particular systematic way of computation. To mitigate this problem the technical expert working groups led by [1] reviewed the “Original” algorithm and developed the “Revised” algorithm as a new standard way that could be consistently used over time and across Countries. The revision encompassed: Removing inconsistently collected data; avoiding categorizing women with missing data as having unmet need; simplifying classification of unmet need for spacing and limiting by inclusion of “wantedness” of current pregnancy/ last birth; shortening duration for consideration of postpartum amenorrheic status from five to two years; standardizing calculation of infecundity; and, explicitly providing directions on handling inconsistencies during DHS exercise. Similarly, to conceptualize the algorithm for deriving estimates of unmet need for contraception, let yi encompass all women who are married, fecund, within the reproductive age from 15 to 49 years and their respective contraceptive need and demand, xj be any women who is a contraceptive method nonuser, xk be a woman who is pregnant or postpartum amenorrheic, xl be “wantedness” of current pregnancy or last birth (*did not want at all, wanted later, missing data*), xm is fecund not pregnant or postpartum amenorrheic, and xn is “wantedness” of children (*wants no more, wanted child in next 2 years or undecided of timing, missing data*). The general formulae for generating group can be given by;


$$\begin{array}{c} G_{\text{2}}=\sum_{i}^{n}\frac{xj\cap (xk\cup xl\cup xm\cup xn)}{yi} \end{array}$$

where i = 1 to n, j = 0, k and m = union of condition 1–2,while l and n = union of condition 1–3

This study improves the estimation by including an additional criteria that incorporates husbands’ virility loss attendant to genetic, ill-health, social or lifestyle factors which are derived by examining variable v3a08x (“Reasons for not using: Others”) as suggested in fertility literature. This inclusion arises since those women who are not using contraception and do not want another childbirth may be doing so because they are aware of their husband’s infecundity, and perceive they are not at risk of pregnancy, hence a justification for exclusion from having unmet need for contraception. To operationalize this proposition that generates a modified “Revised” algorithm, let xp =1 if reason for contraceptive non-use is “Others” and specified verbatim response can be classified as a husband virility issue attendant to any of the following conditions: Genetic factors (azoospermia, aspermia, and undescended testicles); health problems (diabetes, testicular or prostate cancer, sexually transmitted infections, and chronic disease); social problems (stress, depression, and hormonal imbalance) or lifestyle factors (habituation, asexual, and trauma). Further, to concretize this aspect of husband virility issues, a constraint on practice of coital interaction within the month is incorporated with options of befitting being sexually active. Using this consideration, the modified formulae for generating estimates of unmet need for contraception can be re-written as;


$$\begin{array}{c} G_{\text{2}}=\sum_{i}^{n}\frac{xj\cup xp\cap (xk\cup xl\cup xm\cup xn)}{yi} \end{array}$$

where i = 1 to n, j = 0, p = 1–4, k and m = union of condition 1–2, while l and n = union of condition 1–3

## Materials and methods

This study utilized the “Individual Women Data (Individual Recode - IR)” derived from the sixth cycle of the DHS conducted nationally in Kenya between May and October 2014. This data encapsulated all eligibly selected and successfully interviewed women of reproductive age totaling to 31,079 [22]. Globally, all the DHS materials are hosted for public use by the DHS program and instructions for access to use the materials is available at https://dhsprogram.com/data/Access-Instructions.cfm upon registering and approval.

Measurement of unmet need for contraception requires consideration of multifarious variables as explained in the DHS standard record [23]. Considerably, during the DHS exercise at data collection level, the prior trained research enumerator is allowed to record verbatim responses from the women of reproductive age who qualify to answer Section 7: Fertility preferences. Amongst them, there is a sample that qualifies to answer question No. 709 (*You have said that you do not want a/another child soon. Can you tell me why are you not using a method to prevent pregnancy? – Probe- Any other reason)*. In an instance where the respondent provides a reason that is not included in the pre-coded list of reasons, the enumerator probes and documents the verbatim response in the provided spaces. During analysis, the DHS core team at the country level performs the preliminary data analysis, which, amongst other steps, is to align the variables to conform to the required DHS template for purposes of ease of use, comparability and replicability. Additionally, the core team peruses the verbatim responses, for example Question No. 709, and if the reason is genuinely unique and conforms to fertility issue, they create a binary variable v3a08x (“Reasons for not using: Others”) with values 1 if true and a 0 if otherwise.

Building on the “Revised” algorithm for estimating unmet need for contraception, this study paid close attention to variable v3a08x *(“Reasons for not using: Others”)* whose outcome is to generate the modified “Revised” algorithm. This study probed further on genuine reasons and examined those befitting husbands’ loss of virility besides having sexual activity in the last four weeks derived from variable v536. This argument is corroborated by fertility literature which states that globally, one percent of male population have azoospermia [11] and about one in every seven couples are unable to procure pregnancy notwithstanding continual practice of uncontrolled coital interaction [15]. For the purpose of this study and specifically for computing unmet need for contraception, variables that measured contraception need, demand and background characteristics were extracted. Reference is made to the excerpt of the Demographic and Health Survey tool containing questions and skip routines as presented by [1] while all other attendant variables are documented by [23]. Using the context of “exposure to contraceptive needs” that encapsulates all women of reproductive age who are married, fecund, pregnant, in postpartum amenorrheic or infecund/ menopausal, those who qualified and hence adopted as this study’s sample were 8710. Since this study used the secondary data, it therefore must comply with the instructions provided for “data access” at DHS program and hence ride on the already approved ethical considerations of the 2014 KDHS.

This study used Stata ® version 18 during the analysis and the terms “Modified” and “Revised” refers to estimates of total unmet need for contraception generated using the modified “Revised” and “Revised” algorithms, respectively. Considering the “Modified” estimates as reference point, corresponding differences yields values that are positive, zero or negative, which are equivalently interpreted as “underestimation”, “no change” or “overestimation.

It is noteworthy that, a careful documentation of variables considered for use in computation of a composite variable on contraception needs has been done and, the attendant released DHS data for public use meets the required quality threshold. For the purposes of further analysis adopted by this study, a keen focus was taken while considering variables for documenting reasons for contraceptive nonuse. Other consideration was done with respect to attendant variables including coitus in last one month, having given birth within the last five years and not in the postpartum amenorrheic status, which serves as a good enough proof of fecundity.

## Results

These results in Table 1 indicate that the total unmet need for contraception among exposed women of reproductive age in Kenya obtained using the modified “Revised” algorithm is estimated at 16.4 percent. Further, using the “wantedness” constraint, this proportion is dichotomized such that 8.6 percent are classified as having unmet need for spacing and 7.7 percent for limiting childbirths, respectively. Complementing the exclusion of overlooked husbands’ issues, the total unmet need obtained using the “Revised” algorithm among married women of reproductive age in Kenya is 17.5 percent, comprising of respective 9.2 and 8.3 percent for unmet need for spacing and limiting childbirth [22]. As expected and confirmed, the “Modified” estimates are lower than the “Revised” estimates, with the former being 1.15 percentage points higher, which translates to overestimation by 6.6 percent. Similarly, “Modified” estimates are lower than “Revised” estimates of unmet need for contraception for both spacing and limiting childbirth, an implication of overestimation by 0.58 and 0.56 percentage point equivalent to respective 6.4 and 6.8 percent.

**Table 1:** Modified and revised estimates of unmet need for contraception.

	Estimates				Difference (M-R)	
	Modified (M)		Revised (R)		Percentage point	Percentage change
Total respondents (n)	**Count**	**Percent**	**Count**	**Percent**	–	–
	8710	100.0	8710	100.0		
Total unmet need	1423	16.4	1523	17.5	−1.15	−6.6%
Unmet need for spacing	749	8.6	800	9.2	−0.58	−6.4%
Unmet need for limiting	674	7.7	723	8.3	−0.56	−6.8%

**NB:** Modified= Author (2026); Revised= Bradley et al., (2012).

The modified estimates for unmet need for contraception are further segregated by various demographic and socioeconomic characteristics of married women of reproductive age in Kenya and the results are shown in Table 2. The results distributed by age categories shows that the estimates are highest at 21.6 percent and lowest at 14.0 percent among women aged 15–19 and 25–29 years, respectively. Overall these estimates are noticeably lower compared to those computed using the “Revised” algorithm in all age categories. The results further indicate that “Modified” estimates of unmet need for contraception declined with increasing levels of education, with the “Modified” estimates being markedly lower an implication of overestimation.

**Table 2:** Differentiated estimates of total unmet need by married women characteristics.

	Total unmet need estimates		Difference (M-R)	
	Modified (M)	Revised (R)	Percent point	Percentage change
Sample respondents (n)	1423	1523		
Age				
15-19	21.6	23.2	−1.6	6.7%
20-24	17.3	18.9	−1.5	8.1%
25-29	14.0	14.9	−0.9	5.8%
30-34	15.2	15.9	−0.8	4.8%
35-39	17.8	18.5	−0.7	4.0%
40-44	20.0	21.8	−1.8	8.2%
45-49	14.9	16.7	−1.9	11.2%
Education				
No education	26.9	27.9	−1.0	3.6%
Primary	17.8	19.2	−1.4	7.3%
Secondary and above	11.6	12.4	−0.8	−7.1%
Wealth quintile				
Lowest	27.4	28.6	−1.2	4.3%
Second	21.1	23.1	−2.0	8.7%
Middle	16.3	17.1	−0.8	4.9%
Fourth	11.3	12.0	−0.7	5.6%
Highest	9.9	11.0	−1.1	10.2%
Residence				
Urban	12.4	13.4	−1.0	7.3%
Rural	18.9	20.2	−1.3	6.2%
Region				
Coast	20.0	20.7	−0.7	3.3%
North Eastern	30.0	30.0	0.0	0.0%
Eastern	11.6	12.4	−0.8	6.2%
Central	7.9	8.8	−0.9	10.5%
Rift valley	20.1	20.8	−0.7	3.3%
Nairobi	9.4	11.1	−1.7	15.1%
Western	19.2	20.8	−1.5	7.4%
Nyanza	20.9	23.2	−2.4	10.3%

**NB:** Modified= Author (2026); Revised= Bradley et al., (2012).

Additionally, the results show that unmet need are highest amongst women in the lowest wealth quintile and the “Modified” estimates are lower, denoting an overestimation ranging from 0.7 to 2.0 percentage reflecting a difference of 6.0 and 9.5 percent. The “Modified” estimates for MWRA in their place of residence were considerably lower, with an overestimation by 1.0 and 1.3-percentage point equivalent to 7.9 and 6.6 percent decline. Finally, the “Modified” estimates of unmet need for contraception varied considerably across region, and were markedly lower, an implication of an overestimation ranging from 0.7 to 2.4 percent.

## Discussion, conclusion and recommendation

This study aimed at incorporating the overlooked husbands’ virility issues and generated a modified “Revised” algorithm for estimating unmet need for contraception. Using the 2014 KDHS data, this modified “Revised” algorithm has generated estimates that are 1.15 percent point lower, inferring that modification reduces overestimation by seven percent. Further, this implies that there is a pool of women of reproductive age who are classified as having unmet need but have no unmet need for contraception, thereby corroborating the argument by [10]. These results are complemented by two fertility studies that examined sensitivity of estimates of unmet need for contraception by incorporating genuine contexts as documented separately by [2] and [24]. In the later report, there is overestimation of by 16 percent when women who cited having infrequent coital interaction due to away husband as a reason for contraceptive nonuse were excluded. In the latter report, there is documented overestimation by 23 percent by excluding analyst’s discretion and ability to identify contraceptive non-using women at risk of unintended pregnancy.

These modified revised estimates of total unmet need for contraception were highest among adolescents and lowest among older women, implying existence of an age specific differentiated family planning needs. This observation is corroborated by various fertility studies documenting that, exposed adolescents could be oblivious of challenges encountered during debut of marriage life, being in ‘experimenting stage’, and having frail intention to postpone pregnancy due to inappropriate contraception practice and behaviour. The higher estimates among older women may be attributable to; evidence that 15 percent of couples may stay over one year without conception, indecisiveness in wanting additional children alongside low sexual motivation or physicality [11,12,14,15,25].

Further, this study has revealed that estimates of total unmet need for contraception among married women of reproductive age decreases monotonically with increasing education attainment. These results are corroborated by outcome of a systematic review done by [26] indicating that uneducated married women of reproductive age are two times more likely to have unmet need for contraception unlike their educated counterparts. These results are also in tandem with a study done in Kenya using Performance, Monitoring and Accountability survey which revealed that uneducated women were 2.8 times more likely to have unmet need for contraception compared to educated women [27]. Possibly, the high estimates of unmet need for contraception associated with least educated MWRA could be inadequate information, education and communication related to family planning supplies and services whose efficacy is known to reduce unmet need by 10 percent [6].

Moreover, these results indicate that estimates of total unmet need for contraception among married fecund women of reproductive age decreases with increasing household wealth status. This is complemented by [28] who found that in Ethiopia, married women of reproductive age in lowest and middle quintiles are respectively 5.6 and 2.8 times, more likely to have unmet need for contraception than their counterparts in higher quintiles. Similar results resonates with study in Kenya, which summarized that married women of reproductive age residing in low resourced households had 1.6 times higher estimates of total unmet need for contraception as compared to those in high resourced households [27]. Plausibly, the possible reason could be that, exposed fecund women of reproductive age residing in low resourced families may suffer sustenance context of family planning commodities as compared with their counterparts from affluent backgrounds.

Likewise, this study has found that unmet need for contraception is higher among exposed fecund women of reproductive age residing in rural settings unlike those in urban dwellings. These result are complemented by [29] who in a Pakistan study on socio-demographic determinants of family planning documented that, rural women are 0.9 times more likely to have unmet need for family planning than their counterparts in urban areas. The probable reasons associated with rural setup is the associated accessibility complexities constrained by geographical disposition, besides the permeation of familial and community strictures which undermine women’s autonomy on family planning needs on fertility desires and preferences. Lastly, this study has documented that the lowest and highest estimates of unmet need for family planning among exposed fecund women of reproductive age were in Central and Northeastern provinces, respectively. This observation is supported by results of a study done in Ethiopia which documented that women who lived in less developed regions had higher estimates of unmet need for family planning by five times unlike their counterparts in developed regions [28].

In conclusion, this study sought to refine the estimates of “unmet need” for contraception by generating a modified “Revised” algorithm that incorporated the aspect of husband virility issues as a constraint to the existing estimates. Besides this modification re-categorizing a pool of reproductive age women who are classified as having “unmet need” but genuinely have “no unmet need” for contraception, it has also proven existence of overestimation by seven percent in the current estimates. Complementarily, the study findings corroborates previous fertility studies by other cogent Demographers and Scholars who have presented evidence of overestimation of the current estimates of total unmet need for contraception and similar patterns among MWRA’s background characteristics. This study recommends to Demographers and Family Planning Practitioners to include loss of virility due to genetic, ill health, social and lifestyle factors as part of the prescribed reasons for contraception non-use among reproductive age women in fertility studies.
